# ASCO 2018 highlights: metastatic breast cancer

**DOI:** 10.1007/s12254-018-0450-9

**Published:** 2018-11-20

**Authors:** Gabriel Rinnerthaler, Simon Peter Gampenrieder, Richard Greil

**Affiliations:** 1 IIIrd Medical Department with Hematology and Medical Oncology, Oncologic Center, Paracelsus Medical University Salzburg, Müllner Hauptstraße 48, 5020 Salzburg, Austria; 2 Laboratory of Immunological and Molecular Cancer Research and Center for Clinical Cancer and Immunology Trials, Salzburg Cancer Research Institute, Salzburg, Austria; 3 Cancer Cluster Salzburg, Salzburg, Austria

**Keywords:** MONALEESA-3, SANDPIPER, TONIC, PAKT, PHEREXA

## Abstract

This article reviews the clinically most relevant presentations at the American Society of Clinical Oncology (ASCO) annual meeting 2018 on the topic of metastatic breast cancer. In the randomized placebo-controlled phase 3 trial MONALEESA-3, testing ribociclib vs. placebo in combination with fulvestrant in postmenopausal women or men with hormone receptor-positive (HR+) and HER2-negative (HER2−) advanced breast cancer (ABC), an increase of median progression-free survival (PFS) from 12.8 months to 20.5 months by the addition of the CDK4/6 inhibitor was reported (HR 0.59; *P* > 0.01). Taselisib, an alpha specific PI3K inhibitor, was tested in combination with fulvestrant in pretreated HR+/HER2− ABC patients with PIK3CA mutations in the placebo-controlled phase 3 trial SANDPIPER. PFS was significantly longer (7.4 months vs 5.4 months; HR 0.70, *P* < 0.01) but severe adverse events were more frequent (32% and 9%) in the taselisib group. In triple-negative breast cancer, the AKT inhibitor capivasertib (AZD5363) was combined with paclitaxel as first-line treatment in the placebo-controlled phase 2 trial PAKT. In patients with altered PIK3CA, AKT1 or PTEN, median PFS increased from 3.7 months to 9.3 months (HR 0.30; two-sided *P* = 0.01). No treatment effect was shown in the non-altered group. The most common adverse events attributed to capivasertib were diarrhea, fatigue and stomatitis. Results of two phase I trials of trastuzumab antibody-drug conjugates (ADCs) indicated HER2 as a non-oncogenic surface target in breast cancer patients expressing HER2.

## Review

As every year, the American Society of Clinical Oncology (ASCO) annual meeting is a highlight in cancer research. In this short review, the most important presentations from the field of metastatic breast cancer will be highlighted and discussed.

## Luminal breast cancer—targeting CDK 4/6

At this year’s ASCO meeting Dennis Slamon presented the primary results of the randomized phase 3 trial of the CDK4/6 inhibitor ribociclib or placebo in combination with fulvestrant in postmenopausal women or men with hormone receptor-positive (HR+) and HER2-negative advanced breast cancer (ABC) [1, 2]. At least one prior endocrine treatment was allowed. Patients receiving ribociclib had a longer median progression-free survival (PFS) of 20.5 months compared to 12.8 months in the control group (HR 0.59; *P* > 0.01). Furthermore, the overall response rate (ORR) was higher in the investigational arm (32.4% versus 21.5%). In the predefined PFS subgroup analysis, all subgroups were in favor for the CDK4/6 inhibitor treatment. The toxicity profile was in line with previously published trials investigating ribociclib. Grade 3/4 neutropenia was observed in 53% of ribociclib treated patients compared to no patient in the control group. The most common all-grade non-hematologic adverse events occurring in more than 25% of patients were nausea (45% vs. 28%), fatigue (32% vs. 33%), diarrhea (29% vs. 20%) and vomiting (27% vs. 13%). Post baseline QT prolongation (QTcF) of ≥ 500 ms was observed in 2% and <1% in the ribociclib and placebo group, respectively.

Based on this finding, ribociclib combined with fulvestrant may be a new first- or second-line treatment option for postmenopausal women with HR+/HER2− advanced breast cancer.

Several potential mechanisms of resistance to CDK4/6 inhibitors in pre-clinical studies have been published, but the clinical implications of these mechanisms are widely unknown. Nicholas Turner presented results from a substudy of the phase III trial PALOMA 3 [3, 4]. In this randomized phase 3 trial palbociclib and fulvestrant was compared with placebo plus fulvestrant in pretreated postmenopausal women with HR+/HER2- advanced breast cancer. In paired samples of circulating tumor DNA (ctDNA) from baseline and end-of-treatment acquired mutations were investigated potentially leading to treatment resistance. At least one acquired mutation was detected in 28% and 22% of patients in the ribociclib and placebo group, respectively. In both treatment arms acquired mutations in the catalytic subunit alpha of the phosphatidylinositol-3-kinase (PIK3CA; 7% palbociclib and fulvestrant, 10% fulvestrant alone) and estrogen receptor 1 (ESR1; 15% palbociclib and fulvestrant, 9% fulvestrant alone) were most common. Mutations in RB1, the gene encoding the retinoblastoma protein, were infrequent and were only detected in palbociclib treated patients (5%). The authors concluded that a parallel evolution of resistance to fulvestrant and CDK4/6 inhibitors evolves. These findings might influence further treatment strategies for patients resistant to CDK4/6 inhibition.

## Luminal breast cancer—blocking the PI3K pathway

One of the alternated key cancer pathway components of luminal breast cancers are PIK3CA mutations occurring in approximately 40% of patients [5, 6]. Several phosphatidylinositol-3-kinase (PI3K) inhibitors have been developed and clinically tested. Buparlisib, a pan-class I PI3K inhibitor, in combination with fulvestrant has been investigated in two randomized placebo-controlled phase 3 trials (BELLE-2 and BELLE-3 trial) in pretreated postmenopausal women with HR+/HER2− advanced breast cancer [7, 8]. In both trials PFS was significantly longer in the buparlisib arm at the cost of a clinical meaningful increased toxicity. The safety profile of buparlisib did not support its further development in this setting. At this year’s ASCO annual meeting José Baselga presented the primary analysis of the phase 3 trial SANDPIPER investigating taselisib, an alpha-specific PI3K inhibitor, plus fulvestrant vs. placebo plus fulvestrant in HR+/HER2− ABC patients with PIK3CA mutations [9]. Enrolled patients had to be progressing or recurring during or after an aromatase inhibitor treatment, no more than one chemotherapy for the advanced disease was allowed. The investigator-assessed median PFS was significant longer in patients treated with taselisib (7.4 months vs 5.4 months; HR 0.70, *P* < 0.01). Grade 3/4 adverse events were more frequent in the taselisib group occurring in 50% of patients versus 16% of patients in the control arm. Severe adverse events were reported in 32% and 9% in the taselisib and placebo group, respectively. The most frequent adverse events were diarrhea (grade 3/4 12% vs <1%) and hyperglycemia (grade 3/4 11% vs <1%). Because of the modest PFS improvement at the cost of significant toxicity, taselisib will not be further developed.

## Triple-negative breast cancer—targeting altered signaling pathways

The PI3K/AKT/mTOR pathway is frequently activated in TNBC. Clinical activity of the AKT inhibitor ipatasertib in combination with paclitaxel has been shown previously [10]. At ASCO 2018 Peter Schmid presented the results of the randomized phase 2 PAKT trial investigating capivasertib (AZD5363), a highly selective oral small molecule AKT inhibitor, in combination with paclitaxel versus placebo plus paclitaxel as first-line treatment for metastatic TNBC [11]. In the AKT inhibitor group, the investigator-assessed median PFS was numerically longer with 5.9 months compared to 4.2 months in the placebo group (HR 0.74; two-sided *P* = 0.11). The beneficial effect was driven by patients with altered PIK3CA, AKT1 or PTEN genes (20% of patients) with a median PFS of 9.3 vs 3.7 months (HR 0.30; two-sided *P* = 0.01). No treatment effect was observed in the group without alterations in these three genes. The most common adverse events attributed to capivasertib were diarrhea (all grades: 72% vs 27%, grad 3/4 13% vs 1%), fatigue (all grades: 44% vs 26%, grad 3/4 4% vs 0%), rash (all grades: 41% vs 16%, grad 3/4 4% vs 0%) and stomatitis (all grades: 27% vs 14%, grad 3/4 2% vs 0%). Despite the clear efficacy of paclitaxel plus capivasertib in the biomarker selected group, the toxicity profile of capivasertib plus paclitaxel requires further investigation of this treatment combination.

## Triple negative breast cancer—immunotherapy

Breast cancer is a heterogeneous disease and factors influencing the tumor-immune interaction vary between different molecular subtypes [12]. Several early phase clinical trials investigated immune-checkpoint-inhibitor monotherapies in patients with metastatic breast cancer and the most encouraging results were observed in patients with metastatic TNBC when treated in the first-line setting with an ORR of 23% [13]. Several immunological anti-tumor effects have also been proposed for standard treatments [14]. For example, anthracyclines and γ‑radiation can induce immunogenic cell death, and taxanes, platin salts and cyclophosphamide can deplete immune-suppressive regulatory T‑cells (T_regs_). To increase the number of patients benefiting from immunotherapy numerous combination strategies are currently under clinical investigation.

Marleen Kok presented the final stage I response data and first translation results of the adaptive randomized phase 2 TONIC trial of nivolumab after an induction treatment in metastatic TNBC [15]. Up to three prior chemotherapy lines were allowed and an accessible lesion for biopsy or radiation had to be present. Using a non-comparative design, patients were randomized in 5 cohorts: control group (no induction), radiotherapy (3 × 5 Gy), cyclophosphamide (2 weeks 50 mg daily orally), cisplatin (2 × 40 mg/m^2^ IV) or doxorubicin (2 × 15 mg IV). The first dose nivolumab was administered after induction period at week 2 and nivolumab treatment was continued until progression, intolerable toxicity or for 1 year. Blood samples and a biopsy was taken at baseline, at week 2 and week 8. The best ORR was observed in the doxorubicin (ORR 35%, *N* = 17) and cisplatin group (ORR 23%, *N* = 13), while the response was lower in the cyclophosphamide group (ORR 8%, *N* = 12), the radiotherapy group (ORR 8%, *N* = 12) and the control group with no induction treatment (ORR 17%, *N* = 12). In the first translational analysis, an upregulation of pro-immunogenic signatures after cisplatin and doxorubicin treatment were observed at week 2 (post-induction) and week 8 (on nivolumab), each compared to baseline. Furthermore, an increase of T‑cells and T‑cell clonality was observed after a cisplatin or doxorubicin induction phase. The authors announced that the cohort with doxorubicin as an immune induction will be expanded in phase 2 of the trial and cisplatin might also be expanded in the near future based on these encouraging results.

## HER2-positive breast cancer—antibody-drug conjugates and pertuzumab

DS-8201a, an antibody-drug conjugate (ADC) of trastuzumab and duroxtecan, a topoisomerase I inhibitor, was tested in a multiple cohorts phase 1 trial including HER2-positive (*N* = 111) and HER2-low (immunohistochemistry 1+ or 2+ and in situ hybridization negative; *N* = 34) breast cancer patients [16]. ORR and disease control rate (DCR) were 55% and 94% in the HER2-positive and 50% and 85% in the HER2-low breast cancer cohorts, respectively. The most frequent AEs were gastrointestinal or hematologic. Non-hematologic AEs were generally of low grade. Five fatal (5/241) events of interstitial lung disease and pneumonitis were observed in the entire study population and these cases are under review by the adjudication committee. Based on the promising antitumor activity, two phase 3 trials testing DS-8201a versus T‑DM1 (clinicaltrials.gov identifier: NCT03529110) and versus physicians’ choice (NCT03523585) are currently enrolling pretreated HER2-positive ABC patients.

SYD985, an ADC of trastuzumab and duocarmazine, an alkylating agent, was investigated in a phase I expansion cohort study in heavily pretreated patients with HER2-positive or HER2-low metastatic breast cancer. ORR was 33% in HER2-positive (*N* = 50), 27% in HER2-low/HR-positive (*N* = 32) and 40% in HER2-low/HR-negative (TNBC, *N* = 17) patients. The toxicity profile was manageable with AEs mainly of a lower grade. Eye disorders, fatigue, and nausea were the most common AEs. SYD985 is currently tested against treatment of physicians’ choice in a phase 3 trial (NCT03262935).

ZW25 a bispecific antibody binding simultaneously two HER2 epitopes, the trastuzumab binding domain ECD4 and the pertuzumab binding domain ECD2, was investigated in heavily pretreated patients with HER2 expressing cancers. In breast cancer patients (*N* = 20) ORR was 33%. Despite one fatigue grade 3 all observed AEs were grade 1 or grade 2 in the entire study population (*N* = 42) and infusion reactions were the most common side effects.

In the randomized phase 3 trial PHEREXA the addition of pertuzumab to trastuzumab plus capecitabine was investigated in HER2-positive metastatic breast cancer patients progressing during or after a trastuzumab-based therapy. As previously reported, in the dual HER2 blockade group, median PFS was not statistically significantly increased from 9.0 months to 11.1 months (HR 0.82; 95%CI 0.65–1.02; *P* = 0.07) [17]. In the final OS analysis, presented at ASCO 2018, median OS was increased from 28.1 months in the control group (*N* = 224) to 37.2 months in the pertuzumab group (*N* = 228; HR 0.76, 95%CI 0.60–0.98) [18]. Due to hierarchical testing, this was not statistically significant. Based on this trial results, T‑DM1 remains the second-line treatment standard. However, despite the formal negative results of the PHEREXA trial, a clinically relevant activity of trastuzumab plus pertuzumab in pretreated patients can be assumed.

## Conclusion

Results of clinical trials and translational research projects presented at the ASCO annual meeting 2018 extend our knowledge of the treatment and biology of metastatic breast cancer. Furthermore, one of the presented studies will potentially influence our daily clinical practice: based on the findings from MONALEESA-3, ribociclib combined with fulvestrant may be a new first- or second-line treatment option for postmenopausal women with HR+/HER2− advanced breast cancer.
